# Clinical, immunologic and virologic outcomes of children and adolescents receiving second line anti-retroviral therapy in two referral hospitals in Addis Ababa, Ethiopia

**DOI:** 10.1371/journal.pone.0249085

**Published:** 2021-03-30

**Authors:** Endashaw Tekliye, Tinsae Alemayehu, Tigist Bacha

**Affiliations:** 1 Sabiyan General Hospital, Dire Dawa, Ethiopia; 2 American Medical Center, Specialty Clinic for Infectious Diseases and Travel Medicine, Addis Ababa, Ethiopia; 3 St. Paul’s Hospital Millennium Medical College, Addis Ababa, Ethiopia; 4 College of Health Sciences, Addis Ababa University, Addis Ababa, Ethiopia; 1. IRCCS Neuromed 2. Doctors with Africa CUAMM, ITALY

## Abstract

**Background:**

Ethiopia launched free access for antiretroviral therapy in 2005. The number of patients on second line antiretroviral treatment has increased with each passing year. The objectives of this study were to describe the clinical, immunological and virologic outcomes of children and adolescents receiving second line anti-retroviral therapy in two referral hospitals, Yekatit 12 and Zewditu Memorial Hospitals, in Addis Ababa, Ethiopia.

**Methods:**

This was a hospital based retrospective cohort study conducted among children and adolescents aged 18 years and less and receiving a second line antiretroviral drugs. Data was collected using structured questionnaires. Means and percentages were used for nominal variables. Statistical analysis was made using statistical software–SPSS 23.0. Kaplan Meier analysis, long rank test and multivariate Cox proportion model were used to identify factors affecting survival.

**Results:**

A total of 75 children and adolescents were studied with a mean age of 13.28 years (SD: 4) with a mean treatment period on second line regimens of 35.2 months (SD: 21.8 months). Forty-eight participants were experiencing successful measures (in all three parameters) for their second line anti-retroviral treatment. Ten had virologic treatment failure while seven had died. Both treatment failure and death rates were higher within the first two years of treatment. Poor treatment adherence (Adjusted hazard ratio: 5.1 (95% CI: 1.1–23.2; p-value = 0.02)) and advanced World Health Organization clinical stage at start of the second line antiretrovirals (Adjusted hazard ratio: 7.51 (95% CI: 1.35–18.02; p-value = 0.002)) correlated significantly with survival of children and adolescents receiving treatment.

**Conclusions:**

The study describes clinical, immunological and virologic outcomes of second line antiretroviral treatment in a pediatric cohort under care in two hospitals in Addis Ababa, Ethiopia. Poor adherence and pre-treatment advanced clinical stages were predictors of survival.

## Introduction

Human immunodeficiency virus (HIV) continues to challenge health care in sub-Saharan Africa [1]. A 2019 summary of data on the global HIV epidemic by the World Health Organization (WHO) showed 38 million people were living with HIV (children aged younger than 15 years being 1.8 million) with 690,000 deaths due to HIV-related illnesses in the same year (children aged younger than 15 years: 90,000) [2]. Seven in ten individuals living with HIV reside in sub-Saharan Africa [3]. More than 720,000 Ethiopians were living with HIV in 2017 (1.16% of the population) with children aged 14 years or less accounting for 83,579 [4].

Antiretroviral treatment (ART) has prevented millions of deaths and contributed to better life expectancy and global economy. But 41% of people living with HIV were not receiving ART globally in 2017 [5]. Only a quarter of children aged 14 years or less in need of ART in Ethiopia were receiving treatment [4].

Options for second line ART (better explained as a second regimen of antiretroviral treatment) are limited in low resource settings [6]. Pediatric treatment options are further restricted due to lack of approval, availability of formulations compatible for use by children and fixed-dose combinations. This has been shown to contribute for persistence of children on failed first line antiretroviral drugs and subsequent failure of second line regimens after a brief treatment duration [7, 8].

Identifying the success of second line ART regimens and addressing short-comings using intensive counseling, adherence support and improving facilities for virologic testing prevents premature switches to difficult-to-access third line regimens [9].

Little is known about the success of second line ART regimens initiated for children in Ethiopia. A study reporting from a pediatric cohort at Tikur Anbessa hospital of Addis Ababa, Ethiopia (mean age: 16 years) showed high rates of virologic failure (18%) and marked delays in identifying a failed first line regimen (71 months) and initiating second line ART (close to 5 months) [7]. Many others have reported from predominantly adult study populations, leaving insufficient data on children and adolescents [8, 9]. The objectives of the study were to describe clinical, virologic and immunological outcomes of children and adolescents at pediatric HIV clinics in two referral hospitals in Addis Ababa, Ethiopia. In doing so, the envisaged outcomes are to broaden the knowledge on this area of anti-retroviral treatment and care and to improve the quality of available clinical data to inform health workers and policy makers.

## Materials and methods

### Study design and setting

This was a hospital based retrospective cohort study conducted among children and adolescents aged 18 years and less and receiving second line antiretroviral drugs. The study was conducted from 1^st^ March to 30^th^ June 2019.

The two study sites are both government hospitals. The pediatric HIV clinic of Yekatit 12 Hospital and Medical College gives care to 430 children and adolescents with 78 receiving second line ART regimens. The pediatric HIV clinic at the Zewditu Memorial Hospital gives care to 50 children and adolescents on second ART regimens and is one of the foremost HIV treatment and care centers in Ethiopia.

All children and adolescents receiving second line ART formulations at the two study sites and aged 18 years or less were invited for participation.

### Data collection methods

Data was collected from patients’ charts’ using structured paper forms consisting of sociodemographic, pre- and post-treatment clinical, virologic, immunologic and therapeutic variables. Participants had follow-up periods in their respective clinics which ranged from January 2009 to June 2019. Data was collected by pre-trained intern physicians (final year of Doctorate of Medicine or M.D. study program) and nurses serving in the two study sites. Data quality was ensured by three hour training sessions prior to data extraction for data collectors and periodic follow-ups by the principal investigator.

Treatment adherence was determined as good (at least 95% of pills taken since last visit), fair (85–95%) and poor (less than 85%) based on national treatment guidelines [4]. Clinical failure was diagnosed by new or recurrent clinical event indicating advanced or severe immunodeficiency (WHO clinical stages 3 and 4 with exception of Tuberculosis) after 6 months of successful treatment. Immunologic failure was defined as:

- Ages < 5 years: Persistent CD4 levels < 200/mm^3^ or CD4% < 10%;
- Ages 5–10 years: Persistent CD4 levels < 100/mm^3^ and
- Ages 10–18 years: CD4 < 250/mm^3^ after clinical failure or persistent CD4 < 100/mm^3^

Virologic failure was taken as plasma viral loads of above 1000 copies/ml on two consecutive viral load measurements three months apart with enhanced adherence support after the first record of viral load above 1000 copies/ml, and after taking at least six months of second line ART [4].

### Statistical analysis

After data was collected and recorded using paper forms, it was transferred to the Statistical Package for Social Sciences (SPSS) version 23.0 software for analysis. Kaplan-Meier analysis, log rank test and multivariate Cox proportion model were utilized to predict survival curves.

Statistical results are expressed using medians and inter-quartile ranges while regression analyses reported using adjusted hazard ratios, confidence intervals and p-value whose significance was taken as less than 0.05. We selected variables for testing based on findings from similar previous studies [6, 8–11, 16, 18–20, 24]. All p-values greater than or equal to 0.001 are reported as they are while p-values less than 0.001 are expressed as p < 0.001.

### Ethical considerations

The study protocol was reviewed and cleared by the Research and publications’ ethics committee of the Department of Pediatrics and child health, College of health sciences, Addis Ababa University. All data were fully anonymized before investigators accessed them.

## Results

A total of 75 participants took part in the study. Of these, 34 were on follow-up at Yekatit 12 Hospital and Medical College and 41 at Zewditu memorial hospital. The mean age of participants was 13.28 years (SD: 4; Range: 3–18 years). Forty-one were males and 34 females. The differing clinical backgrounds of participants and their caregivers/parents is presented in Table 1.

**Table 1:** Socio-demographic features and clinical backgrounds of participants and their parents/caretakers.

Variable	Frequency
**Age of participants**	
5 years and less	2
5–10 years	14
10–18 years	59
**Status of parents at time of study**	
Both parents alive	17
Both parents deceased	25
One parent alive	20
Unknown	11
**Primary caregiver**	
Both parents	15
Mother	10
Father	4
Orphan and cared for by guardians	39
Orphan and self-caring	6
Undocumented	1
**HIV serostatus of primary caregiver**	
Positive	27
Negative	2
Unknown/Untested	46
**Literacy of parent/caregiver**	
Illiterate	14
Able to write and read	14
Completed secondary school education	10
Completed diploma/Bachelor’s degree training	32
Completed Masters’ or above level of training	4
Undocumented	1
**Employment of parent/caregiver**	
Employed	48
Unemployed	26
Undocumented	1

**Fig 1:**
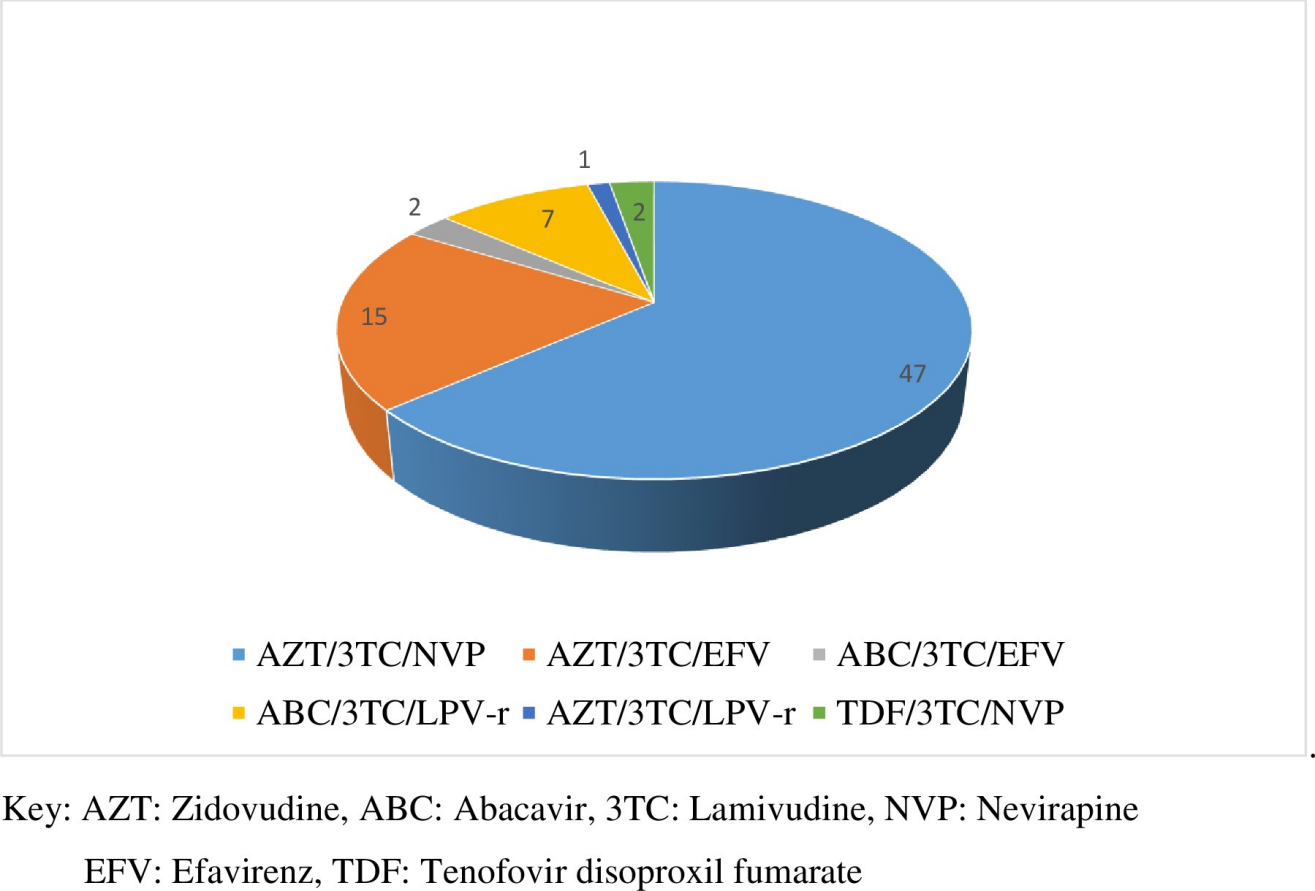
Type of first line ART regimen started for patients before switch to second ART.

The mean duration on first line ART prior to shifting to a second line antiretroviral drugs (ARVs) was 78.7 months (SD: 29.01). There was a mean delay of 12.56 months (SD: 8.23) between confirmation of failure of first line ART and initiation of second line ARVs. From 67 children and adolescents with complete follow-up records, 25 had intermittent losses to follow-up while on first line treatment while 26 had never missed a follow-up appointment. Data on quality of follow-up visit frequency was unknown for the remaining 16. The component antiretroviral drugs of the first line regimens taken by participants is as depicted in Fig 1.

Majority of participants (59/75) had experienced virologic failure for their first line regimens: 22 had both clinical and immunologic failures, 19 had both clinical and virologic failures, with 31 having both immunologic and virologic failures. Eleven patients had fulfilled criteria for clinical, immunologic and virologic failure by the time they were switched to second line ART regimens.

Upon initiating second line treatment, 30 patients were in WHO clinical stage I, 16 in stage II, 18 in stage III and 11 patients in stage IV. Further on, mean CD4 counts at baseline was 257/mm^3^ (median: 184; IQR: 96.8–371.3) and mean viral load was 124,473 copies/ml (median: 35,858; IQR: 10,087–112,232).

**Fig 2:**
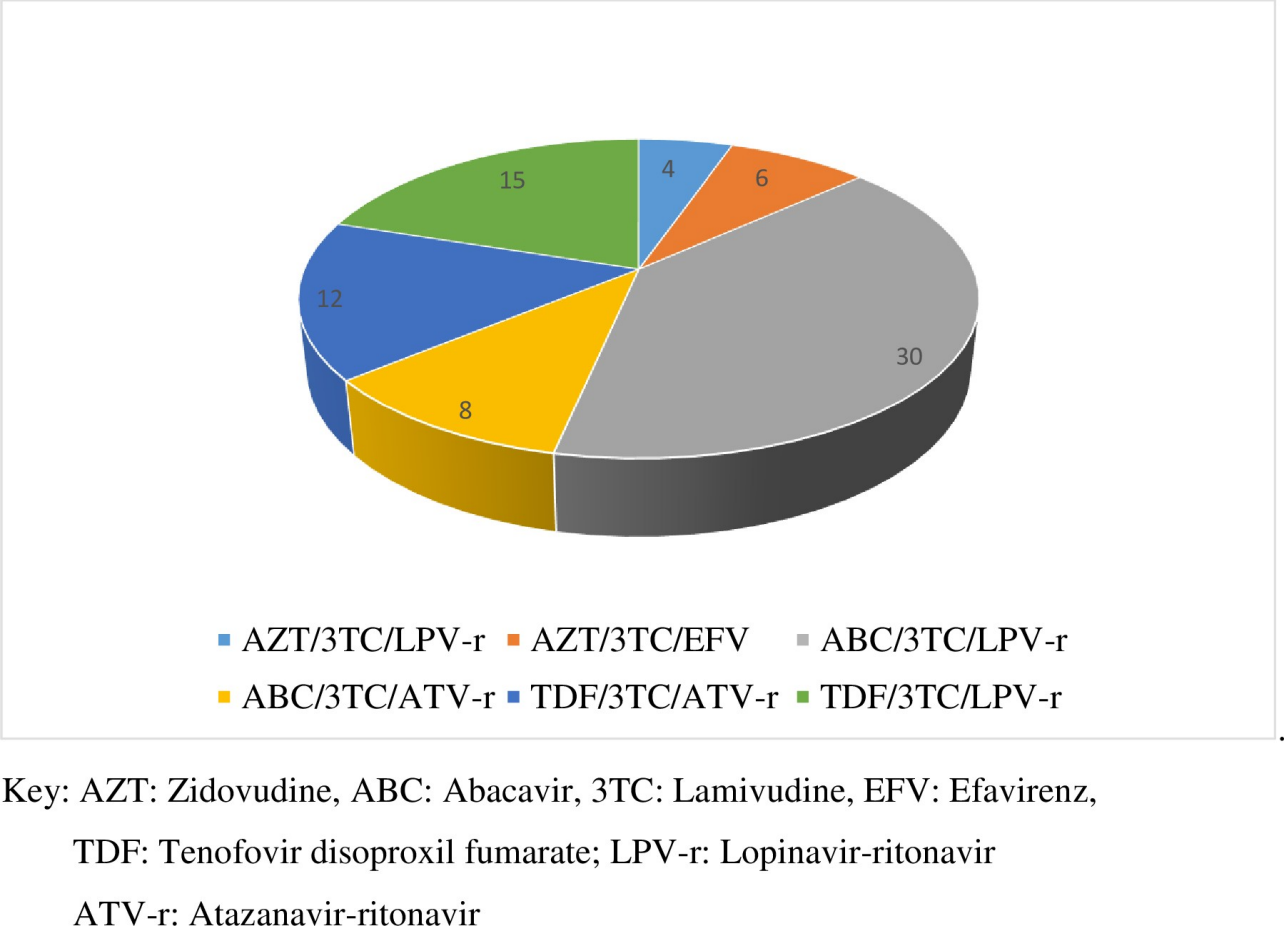
Type of second line ART regimens participants were taking.

Adherence to second line ART regimens was good in 48, poor in 4 and fair in 23 (See component drugs in Fig 2).

The mean second line anti-retroviral treatment period was 35.2 months (SD: 21.8) and the median absence period from follow-up while on second line regimens was 6 months (IQR: 4.50–16.75). Most study participants (54/75) were at disclosure stage III (DS3), while 17 and 3 were at stages DS2 and DS1 respectively.

At the time of conduct of the study, 61 participants were alive, 7 had died after a mean and median duration on treatment of 25.7 and 18 months respectively (4 with a failed second line ARV regimen) and 5 had been lost to follow-up while 2 had transferred out. Ten children and adolescents were experiencing virologic treatment failure (of which two had already been diagnosed accordingly while 8 were diagnosed to have a failed second line ART by investigators). Among 7 patients, criteria to declare treatment failure were not met because they only had a single viral load measurement above 1000 copies/ml and were awaiting enhanced counseling and repeat testing. Of the remaining participants, 48 had suppressed viral loads with 10 having no virologic measures. Fifty-three participants had successful treatments based on immunological measures (CD4 counts) (Table 2).

**Table 2:** Clinical, immunologic and virologic measures during follow-up of participants while on second line ART.

Period of follow-up	Median CD4 cells/mm^3^ (IQR)	Median VL/ml (IQR)	WHO clinical stage			
			I (n)	II (n)	III (n)	IV (n)
Baseline (n = 75)	184 (96.8–371.3)	35858 (10087–112232)	30	16	18	11
6^th^ month (n = 75)	384 (242.3–516.8)	150 (150–952)	62	7	4	2
18^th^ month (n = 53)	535 (354–617)	150 (150–745)	46	6	1	-
30^th^ month (n = 33)	533 (362–673)	150(150–2150)	30	2	1	-
42^nd^ month (n = 22)	682 (624.8–986.3)	337 (132–1161)	21	1	-	-
54^th^ month (n = 15)	631.5 (474–742.5)	150 (150–2517)	15	-	-	-

Key: CD: Cluster differentiation, VL: Viral load, IQR: Inter-quartile range.

None of the patients who had a failed second line treatment had been started on third line ARVs. Resistance testing for anti-retrovirals was unavailable in both study sites.

Testing for CD4 counts and viral loads were sup-optimal. Of the 75 participants, only 48 patients had records of follow-up CD4 testing and 38 had follow-up viral load determinations upon clinical evaluations done after a minimum of 6 months of second line ART (Table 2).

### Survival analysis

**Fig 3:**
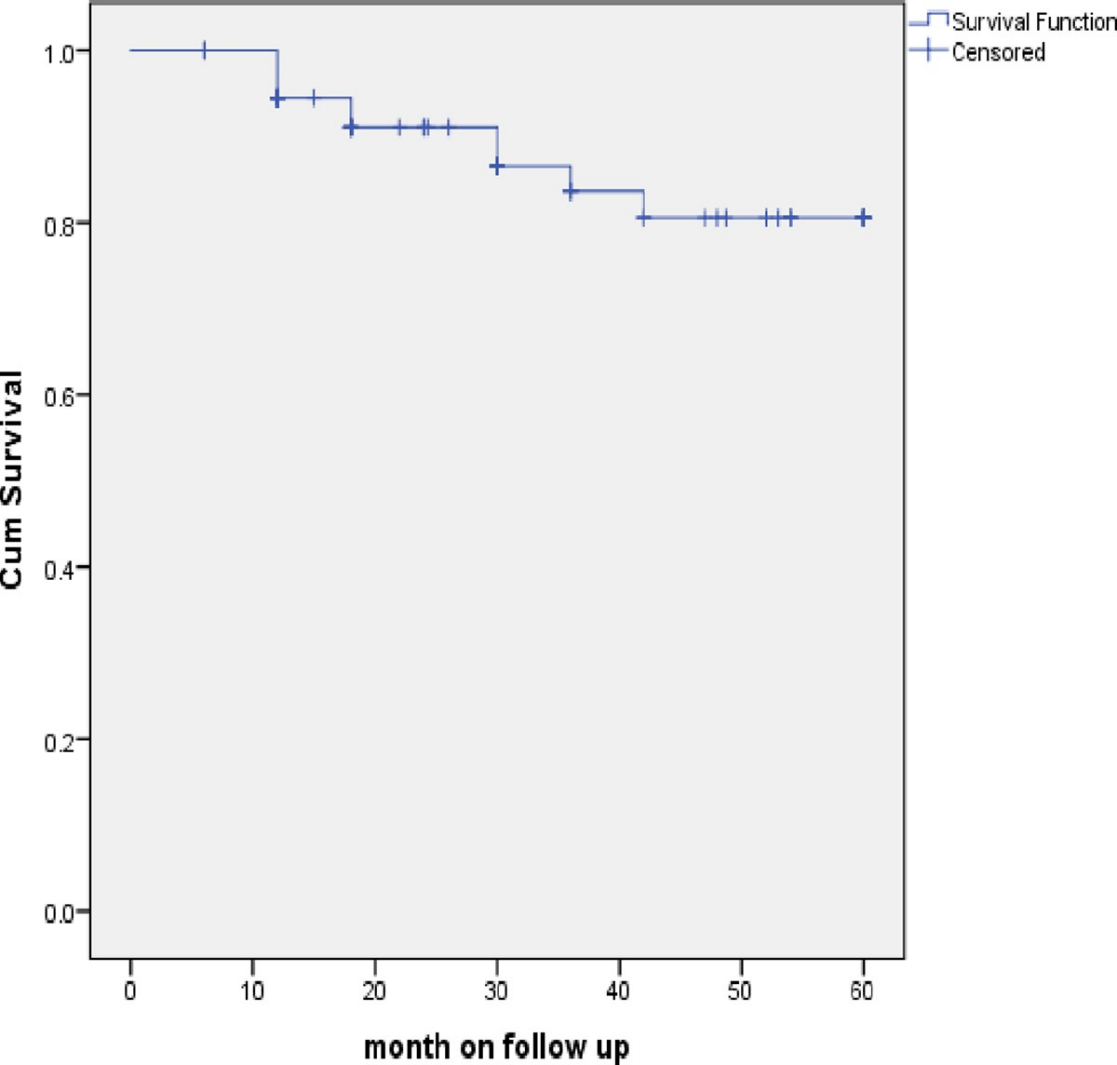
Kaplan Meir survival analysis for study participants while taking second line ART.

The mean survival time using Kaplan Meir analysis is 53.3 months (95% CI: 49.5–57.1). Among those who had developed treatment failure, six out of ten did so in the first 6 to 12 months. Survival was notably good after 30 months of treatment (Fig 3).

Follow up analysis using log rank test showed significant difference in survival time only for two variables: baseline WHO clinical stage upon initiating second line ART and adherence to second line ART drugs.

**Fig 4:**
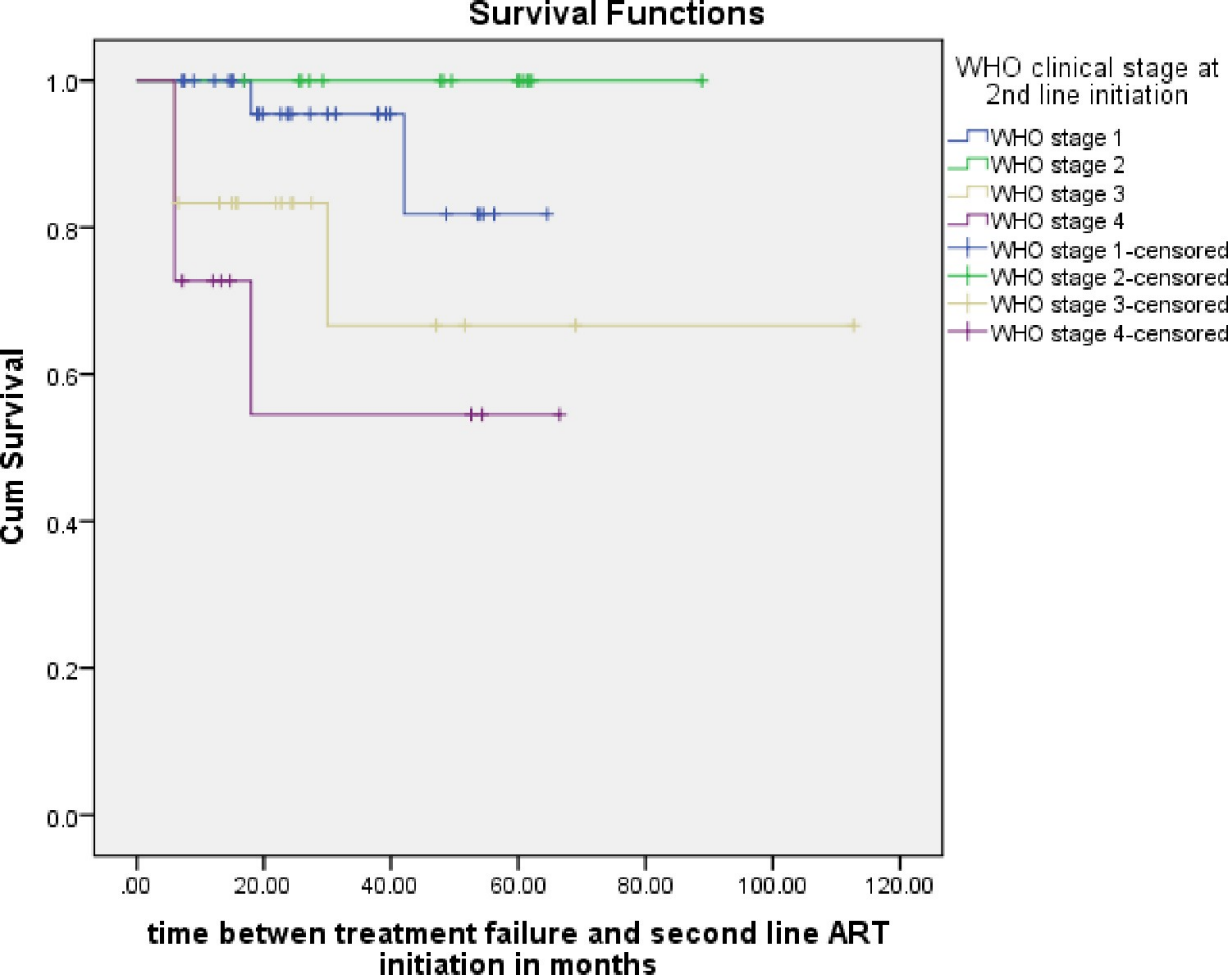
Multivariate cox proportion model: Baseline WHO clinical stages vs survival.

When survival time based on baseline WHO clinical stage was analyzed using multivariate cox proportion model, those with WHO clinical stages I and II fared better than those with advanced stages (III and IV) (Adjusted Hazard Ratio: 7.51; 95% CI: 1.35–18.02; p-value = 0.002) (Fig 4).

**Fig 5:**
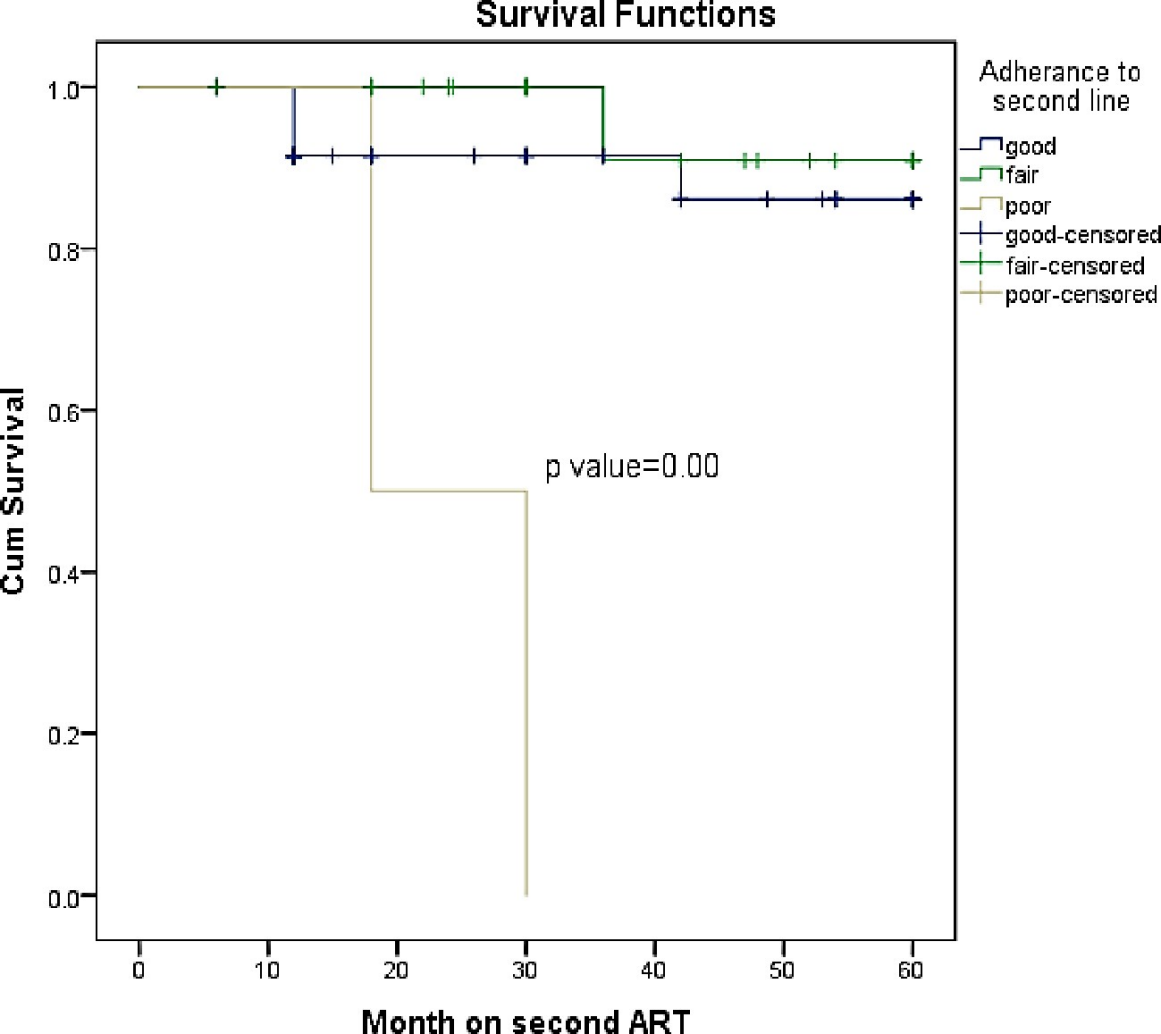
Multivariate cox proportion model: Adherence to treatment vs survival.

Those who have good adherence had a mean survival of 54.9 months (95% CI: 50.7–59.1) versus those who had poor adherence (mean survival: 24 months; 95% CI: 17.2–30.2) (Adjusted Hazard Ratio: 5.1; 95% CI: 1.1–23.2; p-value = 0.02) (Fig 5).

## Discussion

The study showed that second line ART regimens were preceded by delayed initiation, high baseline viral and CD4 counts. There was an approximate gap of one year between conformation of a failed first line ART regimen and initiation of a second one. This fared worse than pediatric cohorts receiving second line ART in South Africa (171 days) and two other studies from Ethiopia (89 days and 144 days) [7, 10, 11]. Postponing early initiation of second line ART regimens increases rates of virologic failure and mortality of children living with HIV [12, 13].

Adherence for initial and second rounds of ARVs was sub-optimal. Recording of adherence is based on parents or caretakers’ report and is at risk for bias in interpretation. Therefore, attempting correlations between adherence and virologic failure among children has proved discrepant [14, 15].

The mean viral load upon initiation of second line ARVs was high. Similar elevated viral set-points were seen among children and adolescents upon being started on second line ARVs in Ethiopia and Nigeria [7, 16]. This mirrors the tolerance of failed first line ARVs. Viral set-points exceeding 100,000 copies/ml at onset of second line ARVs among children and adolescents living with HIV contributes to twice higher mortality [17].

Ten out of seventy-five (13.3%) children and adolescents were experiencing virologic treatment failure (10.6 per 100 person years). These findings were comparable to reports from other low-resource settings in sub-Saharan Africa and south Asia [18–20]. The overall rates may be higher with provision of regular viral load testing. This is of particular importance since virologic failure occurs earlier and provides a more accurate indicator [21]. None of the study’s participants who had a failed second line treatment had been started on third line ARVs.

Out of the ten children with failed treatments, six developed their treatment failure within 6–12 months of initiating the second ART. This was shorter than the 17.6 months it took till failure seen in another pediatric cohort in an Ethiopian referral hospital [7]. Higher pre-treatment viral set-points and WHO clinical stages and poor adherence could be contributing factors for early treatment failure.

Testing for CD4 counts and viral loads were sup-optimal. Strategies to monitor treatment failure differ with the income level of the country. Very few (3%) of pediatric cohorts across sub-Saharan countries barring South Africa receive regular CD4 and viral load monitoring while receiving second line ART [22]. Lack of well-equipped and well-staffed virology labs in study sites may have contributed to deficits in quality of follow-up. Delayed recognition of failed second regimens and referrals for construction of third regimens increases co-morbidities and shortens survival [23].

At the time of data collection, close to 10% (7/75) children had died after a mean duration on treatment of 25.7 months. Four had virologic failure. The mean survival using Kaplan Meir analysis is 53.3 months. Poor prognosis regarding survival correlated with poor adherence and advanced WHO clinical stages upon starting second line ART. Across low- and middle-income countries, adolescent ages, being underweight, a longer period of taking first line ARVs and undetectable drug levels correlated with virologic failure [24]. While adolescence was also seen to be a risk for virologic failure in two other reports from east Africa [7, 19], a short duration on first line ART (less than 2 years) was identified as another risk in a cohort of children in Uganda [19]. Zanoni et al. described that non-nucleoside reverse transcriptase inhibitor (NNRTI) based second line ART for South African children was associated with a higher risk of virologic failure than PI-based ART [25].

The study also showed that 5 out of 75 participants (6.7%) had been lost to follow-up. This mimicked the cumulative incidence of loss to follow-up (LTFU) at the end of 2 years of follow-up among similar cohorts in sub-Saharan Africa (7.1%) versus less than 5% globally, reported by the Collaborative Initiative for Paediatric HIV Education and Research (CIPHER) Global Cohort Collaboration [26].

This study’s analysis of performance of second line ART among the study population showed delayed initiation of treatment, high viral set-points and poor treatment adherence. There was poor implementation of regular viral load and immunologic tests during follow-up. Within this restricted clinical environment, the described virologic failure was in agreement with other pediatric cohorts in low-resource countries. Virologic failure occurred early on into treatment. Poor treatment adherence and advanced pre-treatment WHO clinical stages correlated with survival. Limitations include a small study population, incomplete follow-up testing to further determine performance of treatment and incomplete records of study participants.

## Conclusions

The performance of second line antiretroviral treatments for children and adolescents under care at pediatric HIV clinics in two referral hospitals in Addis Ababa, Ethiopia is described. Improving the depth of knowledge of health workers targeted towards frequent monitoring and early initiation of treatment, improving laboratories’ capacity including their ability to conduct pre-treatment anti-retroviral drug resistance testing and adherence counseling should be priority areas for improvement of HIV care in children and adolescents. We recommend further larger-scale studies to outline the extent of the problem nationwide.

## Supporting information

S1 File (SAV)

## References

[1] Kharsany AB, Karim QA. HIV Infection and AIDS in Sub-Saharan Africa: Current Status, Challenges and Opportunities. *The open AIDS journal* 2016, 10, 34–48. 10.2174/1874613601610010034 27347270 PMC4893541

[2] https://www.who.int/data/gho/data/themes/hiv-aids.

[3] Dwyer-Lindgren L, Cork MA, Sligar A, Steuben KM, Wilson KF, Provost NR, et al. Mapping HIV prevalence in sub-Saharan Africa between 2000 and 2017. *Nature* 570, 189–193 (2019). 10.1038/s41586-019-1200-9 31092927 PMC6601349

[4] The Federal Ministry of Health of Ethiopia: National guidelines for comprehensive HIV prevention, care and treatment February 2018

[5] Forsythe SS, McGreevey W, Whiteside A, Shah M, Cohen J, Hecht R, et al. Twenty years of antiretroviral therapy for people living with HIV: Global costs, health achievements, economic benefits. *Health affairs* 7 2019, 38:7, 1163–1172. 10.1377/hlthaff.2018.05391 31260344

[6] Edessa D, Sisay M, Asefa F. Second-line HIV treatment failure in sub-Saharan Africa: A systematic review and meta-analysis. *PLoS ONE* 2019; 14(7): e0220159. 10.1371/journal.pone.0220159 31356613 PMC6663009

[7] Alemayehu T, Abebe W. Second line anti-retroviral therapy failure in a pediatric cohort of an Ethiopian tertiary hospital: a retrospective observational study. *Sci Rep* 2020; 10, 8699. 10.1038/s41598-020-65714-6 32457309 PMC7250842

[8] Zenebe Haftu A, Desta AA, Bezabih NM, Bayray Kahsay A, Kidane KM, Zewdie Y, et al. Incidence and factors associated with treatment failure among HIV infected adolescent and adult patients on second-line antiretroviral therapy in public hospitals of Northern Ethiopia: Multicenter retrospective study. *PLoS ONE* 2020, 15(9): e0239191. 10.1371/journal.pone.0239191 32986756 PMC7521713

[9] Alene M, Awoke T, Yenit MK, Tsegaye AT. Incidence and predictors of second-line antiretroviral treatment failure among adults living with HIV in Amhara region: a multi-centered retrospective follow-up study. *BMC Infect Dis* 19, 599 (2019). 10.1186/s12879-019-4243-5 31288748 PMC6617674

[10] Davies MA, Moultrie H, Eley B, Rabie H, Van Cutsem G, Giddy J, et al. Virologic failure and second-line antiretroviral therapy in children in South Africa—the IeDEA Southern Africa collaboration. *J Acquir Immune Defic Syndr.* 2011;56(3):270–278. 10.1097/QAI.0b013e3182060610 21107266 PMC3104241

[11] Haile GS, Berha AB. Predictors of treatment failure, time to switch and reasons for switching to second line antiretroviral therapy in HIV infected children receiving first line anti-retroviral therapy at a Tertiary Care Hospital in Ethiopia. *BMC Pediatr* 2019; 19, 37. 10.1186/s12887-019-1402-1 30696412 PMC6352354

[12] Khan S, Das M, Andries A, Deshpande A, Mansoor H, Saranchuk P, et al. Second-line failure and first experience with third-line antiretroviral therapy in Mumbai, India. *Global Health Action* 2014; 7:1, 10.3402/gha.v7.24861 25084835 PMC4119292

[13] Rohr GK, Ive P, Horsburgh R, Berhanu R, Shearer K, Maskew M, et al. Marginal structural models to assess delays in second-line HIV treatment initiation in South Africa. *PLoS One* 2016;11(8): e0161469. 10.1371/journal.pone.0161469 27548695 PMC4993510

[14] Martelli G, Antonucci R, Mukurasi A, Zepherine H, Nostlinger C. Adherence to antiretroviral treatment among children and adolescents in Tanzania: Comparison between pill count and viral load outcomes in a rural context of Mwanza region. *PLoS ONE* 2019; 14(3), e0214014, 10.1371/journal.pone.0214014 30897131 PMC6428300

[15] Mehta K, Ekstrand ML, Heylen E, Sanjeeva GN, Shet A. Adherence to Antiretroviral Therapy Among Children Living with HIV in South India *AIDS Behav*. 2016; 20(5):1076–1083. 10.1007/s10461-015-1207-7 26443264 PMC5472452

[16] Okechukwu AA, Amajuoyi FI. First and second line HAART failure in HIV infected Nigerian children at University of Abuja teaching hospital, Nigeria. *J HIV Clin Scientific Res* 2015; 2(2), 049–054, 10.17352/2455-3786.000014.

[17] Mofenson LM, Korelitz J, Meyer WA, Bethel J, Rich H, Pahwa S, et al. The Relationship between Serum Human Immunodeficiency Virus Type 1 (HIV-1) RNA Level, CD4 Lymphocyte Percent, and Long-Term Mortality Risk in HIV-1–Infected Children. *The Journal of Infectious Diseases* 1997; 175, 1029–38. 10.1086/516441 9129063

[18] Boerma RS, Bunupuradah T, Dow D, Fokam E, Kariminia A, Lehman D, et al. Multicentre analysis of second-line antiretroviral treatment in HIV-infected children: adolescents at high risk of failure. *J Int AIDS Soc*. 2017;20(1):21930. 10.7448/IAS.20.1.21930 28953325 PMC5640308

[19] Boerma RS, Kityo C, Boender TS, Kaudha E, Kayiwa J, Musiime V, et al. Second-line HIV Treatment in Ugandan Children: Favorable Outcomes and No Protease Inhibitor Resistance. *J Trop Pediatr.* 2017;63(2):135–143. 10.1093/tropej/fmw062 27634175

[20] Prasitsuebsai W, Teeraananchai S, Singtoroj T, Truong KH, Ananworanich J, Do VC, et al. Treatment Outcomes and Resistance Patterns of Children and Adolescents on Second-Line Antiretroviral Therapy in Asia. *J Acquir Immune Defic Syndr.* 2016;72(4):380–386. 10.1097/QAI.0000000000000971 27355415 PMC4929998

[21] Hailu GG, Hagos DG, Hagos AK, Wasihun AG, Dejene TA. Virological and immunological failure of HAART and associated risk factors among adults and adolescents in the Tigray region of Northern Ethiopia. *PLoS One*. 2018;13(5):e0196259. 10.1371/journal.pone.0196259 29715323 PMC5929526

[22] The Collaborative Initiative for Pediatric HIV Education and Research (CIPHER) Global Cohort Collaboration. Incidence of switching to second-line antiretroviral therapy and associated factors in children with HIV: an international cohort collaboration. *Lancet HIV* 2019; 6: e105–15. 10.1016/S2352-3018(18)30319-9 30723008 PMC7093820

[23] Tsegaye AT, Wubshet M, Awoke T, Alene KD. Predictors of treatment failure on second-line antiretroviral therapy among adults in northwest Ethiopia: a multicentre retrospective follow-up study. *BMJ Open* 2016;6:e012537. 10.1136/bmjopen-2016-012537 27932339 PMC5168604

[24] Suaysod R, Ngo-Giang-Huong N, Salvadori N, Cressey TR, Kanjanavit S, Techakunkakorn P, et al. Treatment Failure in HIV-Infected Children on Second-line Protease Inhibitor-Based Antiretroviral Therapy. *Clin Infect Dis*. 2015;61(1):95–101. 10.1093/cid/civ271 25838288

[25] Zanoni BC, Sunpath H, Feeney ME. Pediatric response to second-line antiretroviral therapy in South Africa. *PLoS One*. 2012;7(11):e49591. 10.1371/journal.pone.0049591 23185373 PMC3502491

[26] CIPHER Global Cohort Collaboration. Outcomes of second-line antiretroviral therapy among children living with HIV: a global cohort analysis. *Journal of the International AIDS Society* 2020, 23:e25477. 10.1002/jia2.25477 32297485 PMC7160415

